# A Comprehensive Comparison Between the Semi-sterile and Sterile Technique for Closed Reduction and Percutaneous Pinning of Pediatric Supracondylar Humerus Fractures

**DOI:** 10.3389/fsurg.2020.594027

**Published:** 2020-10-29

**Authors:** Guo-Qiang Wang, Qing-Feng Wang, Xiao-Dong Wang

**Affiliations:** ^1^Children's Hospital of Soochow University, Suzhou, China; ^2^Department of Orthopedic Surgery, People's Hospital of Ningxia Hui Autonomous Region, Yinchuan, China; ^3^Department of Orthopedics, Children's Hospital of Soochow University, Suzhou, China

**Keywords:** pediatric, sterile, fracture, pinning, recovery, infection

## Abstract

**Background:** Supracondylar humerus fracture is the most common elbow fracture in children, which often requires closed reduction and percutaneous pinning (CRPP) procedure for full recovery. In addition to the traditional sterile technique with full prep and draping, the semi-sterile technique without sterile gowns and drapes has been suggested to be a viable alternative for CRPP.

**Methods:** Here, we performed a retrospective study over a 3-year period to comprehensively evaluate the outcomes of the semi-sterile and the sterile techniques for CRPP in supracondylar humerus fractured patients. Demographic data, fracture type, nerve injury status and the type of preparation technique (semi-sterile vs. sterile) were recorded. Time of preparation and operation, costs and elbow recovery status were compared. Outcomes of the two techniques were compared with bivariate analysis.

**Results:** In a total of 137 patients, we found that the semi-sterile technique could significantly reduce the total operation room usage time (80 ± 13 min vs. 94 ± 12 min, 15% reduction, *P* < 0.001) and costs of CRPP. Specifically, anesthesia and medical waste costs were reduced by 139 RMB (1,736 ± 128 vs. 1,875 ± 197, 7.4% reduction, *P* < 0.001) and 103.0 RMB (14.6 vs. 117.9) per operation, respectively. At the meantime, the infection rate and recovery efficiency (89 ± 10 vs. 91 ± 9 of the Mayo Elbow Performance Score, *P* = 0.352) were almost unchanged as compared to the sterile technique group.

**Conclusions:** Our study suggests that the semi-sterile technique can be used as a cost-effective alternative for CRPP in supracondylar humerus fracture and even other bone-related non-surgical approaches.

**Level of Evidence:** The present study is a retrospective cohort study with a level III of evidence.

## Background

Supracondylar humerus fracture is a type of upper extremity fracture that occurs at the distal end of humerus right above the elbow joint. Despite its low incidence in adults, it is the most common elbow fracture in children, accounting for 50–60% of all elbow fractures (1). Treatment strategy depends on whether the fracture is associated with bone displacement or not. Orthopedic casting is normally enough for non-displaced fractures, while surgery is often the best option for displaced fractures. Based on the level of displacement, the Gartland classification system has divided the supracondylar humerus fracture into three types, which are characterized by displacement free, angulated displacement and complete displacement, respectively (2). The standard treatment for type 2 and 3 fractures is the closed reduction and percutaneous pinning (CRPP) procedure, which is a non-surgical approach that only needs a small incision to put the bone fragment back into place and fix it with a pin under the fluoroscopic guidance. CRPP is traditionally carried out under fully sterile conditions, including the entire upper extremity of the patient and all the personnel that are involved in the surgery. However, the necessity of this fully sterile setting for CRPP has been challenged by several previous studies (3–6). Alternatively, they propose a semi-sterile technique for CRPP that can decrease medical waste and health care costs.

In the present study, we performed a comprehensive comparison of the safety and benefits between the sterile and the semi-sterile technique for CRPP from multiple perspectives to investigate whether the latter could be used as a safe and efficient alternative of the former.

## Methods

Upon approval by the review board, a consecutive retrospective cohort study was conducted on pediatric patients who underwent CRPP for supracondylar humerus fracture over a 3-year period (2017–2019) at Children's Hospital of Bao Tou. All included patients were under the age of 16. Inclusion criteria were patients who suffered type 2 and 3 supracondylar humerus fractures. The only exclusion criterion was the necessity to perform an open approach to achieve reduction of the fracture, where full preparation and draping is mandatory. The need for open reduction is evaluated before any preparation and draping to alleviate the need to convert from a semi- to a full sterile operation. A transition from the sterile technique using full-prep and drape to the previously described semi-sterile technique (3) for all pediatric supracondylar humerus fracture CRPP procedures took place during this period of time. The surgical technique was identical in both groups. Patients were placed under general anesthesia. Closed reduction is achieved by a combination of traction, abduction, and rotation of the injured elbow under direct visualization and the guidance of fluoroscopy. Once fixed, the Kirschner wires were used to percutaneously fix the fracture. Two wires were placed distal to the axillary nerve and gradually advanced to the epiphysis. If needed, a third wire was placed based on the position of the first two to further enhance the stability. The wires were left transcutaneous in place for around 3 weeks after treatment.

For the semi-sterile technique, the operative site was first sterilized by brushing with chlorhexidine and was then placed onto a sterile towel. Surgeons and nurses only wore sterile gloves, but not sterile gowns. Thus, sterile drapes were also not used in the semi-sterile group. After the fracture was stabilized, the elbow was wrapped in sterile cast padding and immobilized in plaster. For the sterile technique, full surgical room preparation and draping of the full upper extremity was applied. Patients' personal information, including age and sex; as well as operative details, such as type of injury, preoperative neurovascular status and operation timing etc. were individually documented.

Patients' characteristics were presented with mean and standard deviation for continuous variables, and frequencies and proportions for categorical variables. Student *t*-test or Wilcoxon rank sum test was used to compare the statistical difference for normally distributed and non-normally distributed continuous data, respectively. A *P*-value of < 0.05 was considered as statistically significant.

## Results

A total of 140 patients were analyzed. Three of them were excluded because of the need of an open reduction and fixation. One hundred thirty-seven of them underwent CRPP: 79 with the semi sterile technique and 58 with the sterile approach. Average age of the patients in the two groups were 6.2 ± 2.3 and 6.1 ± 2.4 years old, respectively; with no statistical difference (*P* = 0.881) (Table 1). There were 48 male and 31 female patients in the semi-sterile group and 39 male and 19 female patients in the sterile group. The patients in both groups were operated with the same delay between injury and operation (*P* = 0.393) (Table 1). Distribution of the types of supracondylar humerus fracture and the preoperative neurovascular status of the patients within the two groups were summarized in Table 1. In general, there were no differences between the groups in neither the distribution of the Gartland type of fracture nor of the associated nerve injuries (Table 1). A total of 15 and 8 patients with nerve injuries were observed preoperatively in the semi-sterile and the sterile groups, respectively; with the radial nerve being the most common injured nerves in both groups (Table 1). It is worth noting that the overall percentage of supracondylar humerus fracture related nerve injury was only around 17% (23/137).

**Table 1:** Patient characteristics.

**Variable**	**Semi-sterile** ** (*n* = 79)**	**Sterile** ** (*n* = 58)**	***P*-value**
Age, mean ± SD	6.2 ± 2.3	6.1 ± 2.4	0.8813
Time before operation (hour), mean ± SD	19.7 ± 7.1	20.7 ± 6.3	0.3925
**Type of fracture: according to Gartland's classification**, ***n*** **(% of total patients)**			
Type 2	38 (48%)	29 (50%)	
Type 3	41 (52%)	29 (50%)	
**Nerve injuries (at diagnosis)**, ***n*** **(% of total patients)**			
Radial	9 (11%)	5 (9%)	
Median	3 (4%)	2 (3%)	
Ulnar	3 (4%)	1 (2%)	

**Figure 1:**
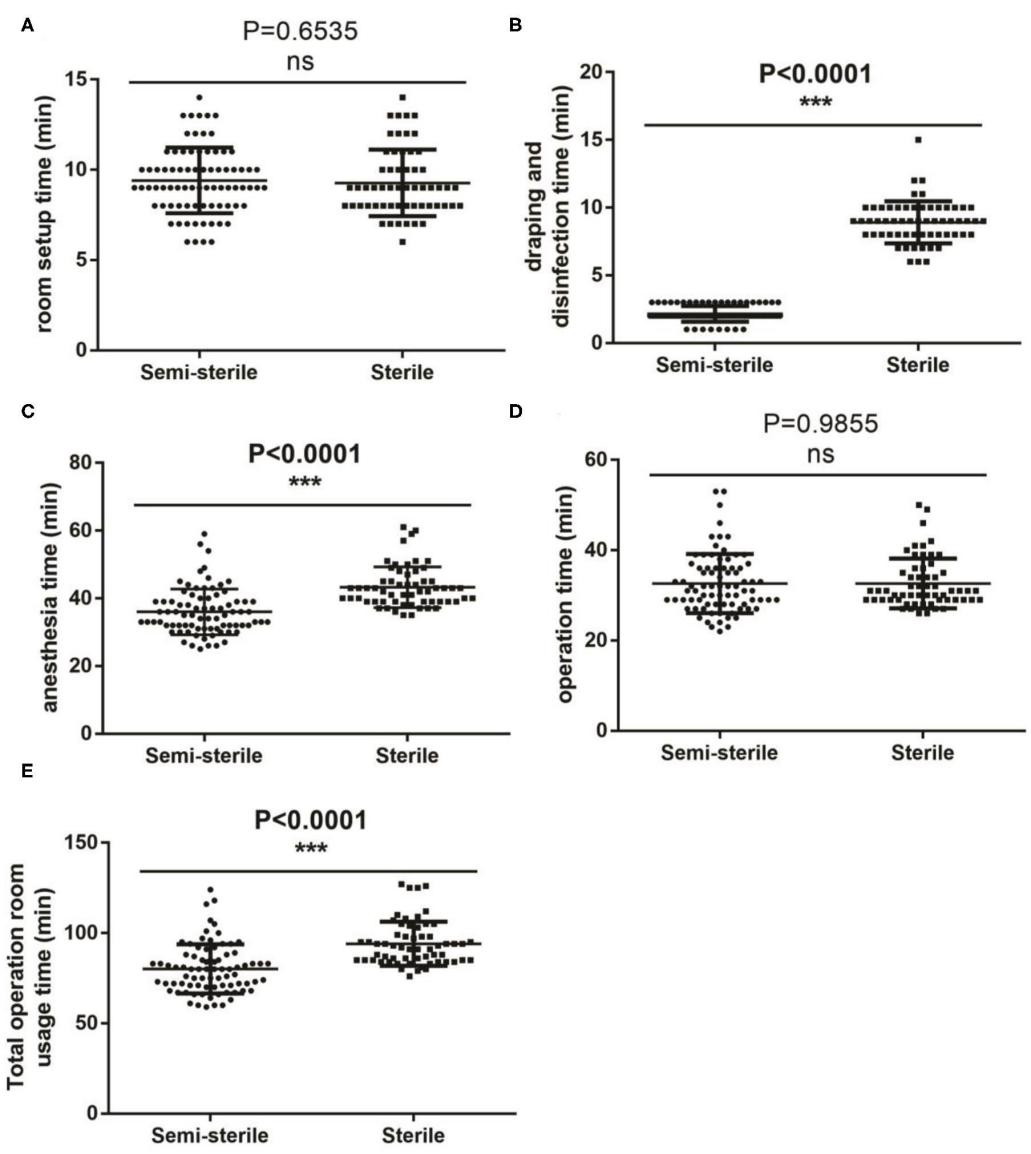
Comparison of the duration of each steps of the procedure between the semi-sterile and the sterile groups. **(A–E)** Comparison of the setup time **(A)**, draping and disinfection **(B)**, anesthesia total time **(C)**, operating time **(D)** and total operating room occupation **(E)** between the two groups. ***Statistically significant; ns, not significant.

**Figure 2:**
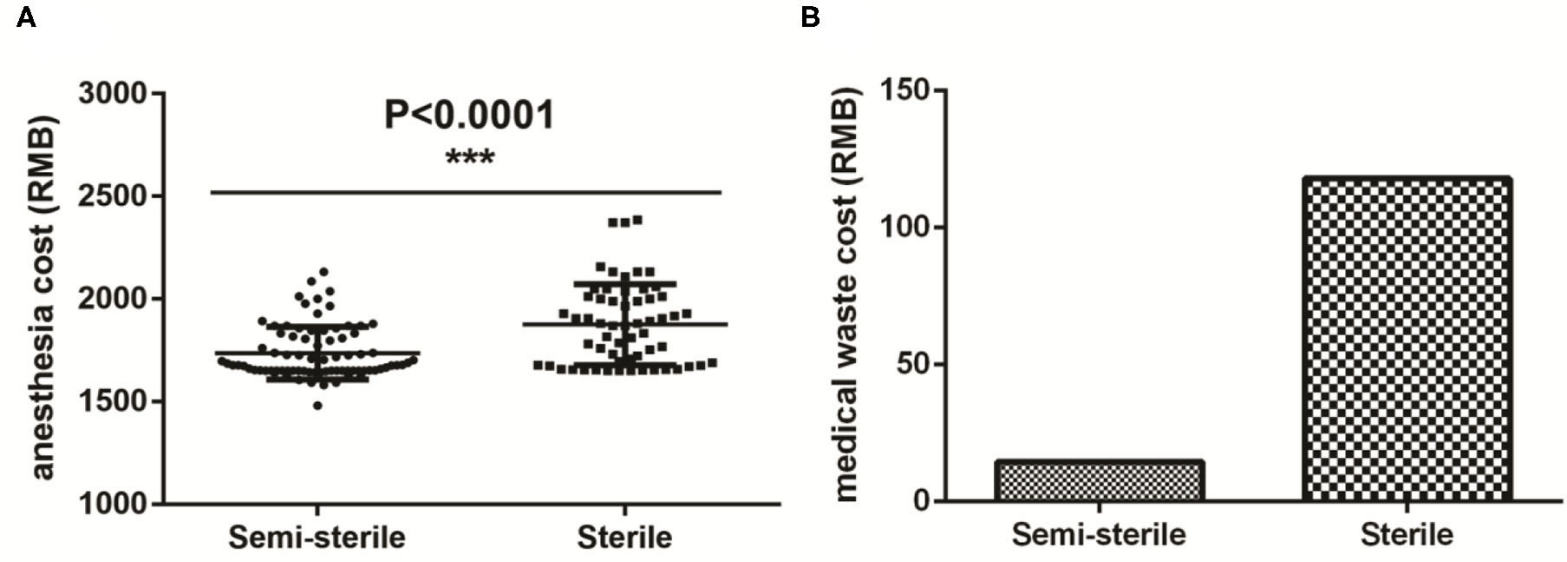
Comparison of the cost difference between the semi-sterile and the sterile groups. **(A,B)** Comparison of anesthesia **(A)** and medical waste **(B)** costs of the two groups. Medical waste cost has the identical value in each group and therefore statistical analysis is not applicable in **(B)**. ***Statistically significant; ns, not Significant.

Figure 1 shows the comparison in the duration between the two groups for various steps of the procedure. As expected, omission of the sterile gowns and drapes in the semi-sterile technique significantly reduced the time for draping and disinfection (Figure 1B) and anesthesia (Figure 1C) by 6.7 (2.2 ± 0.58 vs. 8.9 ± 1.56, *P* < 0.001) and 7.3 (36.0 ± 6.77 vs. 43.3 ± 6.04, *P* < 0.001) minutes, respectively. As a result, a total of 14 min (80 ± 13 vs. 94 ± 12, *P* < 0.001) was saved from the total operation room usage time for each operation (Figure 1E). Procedures that did not involve any sterilization process, such as room setup (*P* = 0.654) and the actual operation (*P* = 0.986) lasted similar time between the two groups (Figures 1A,D); indirectly suggesting that the observed time difference is indeed due to the difference between the two techniques. In term of running costs, significant reductions were observed in both the anesthesia (Figure 2A) and the medical waste (Figure 2B) costs; where 139 (1,736 ± 128 vs. 1,875 ± 197, *P* < 0.001) and 103.3 (14.6 vs. 117.9) RMB were saved for each operation, respectively; which roughly equals to 20 and 15 USD, respectively.

**Figure 3:**
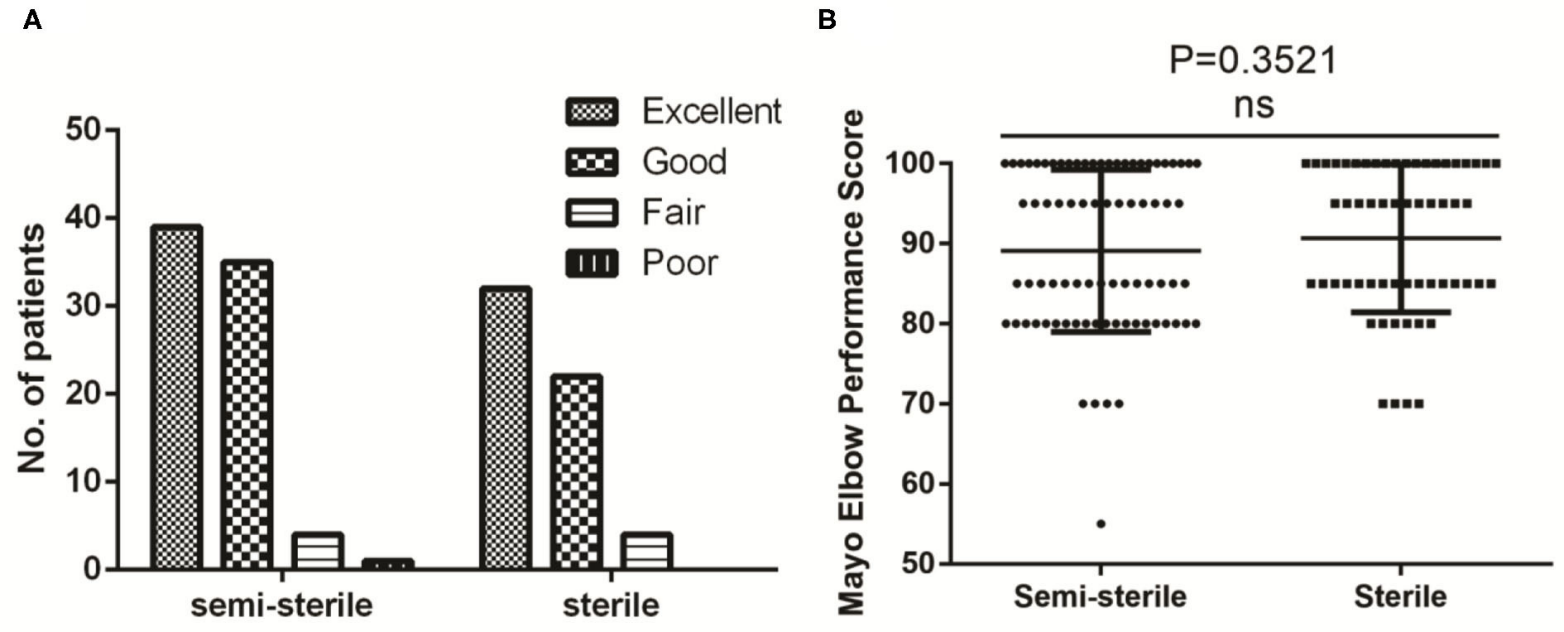
Comparison of functional recovery (**A**, Flynn criteria; **B**, Mayo Elbow Performance Score) between the semi-sterile and the sterile groups.

In term of the treatment outcome, no post-surgery infection was observed in the patients from the sterile group. Only 1 patient in the semi-sterile group suffered superficial infection at the site of operation. For this particular patient, oral admission of antibiotics was given on a regular interval until recovery without pin removal. This patient eventually achieved complete functional recovery according to the Flynn criteria for elbow evaluation (ranked as excellent) and the Mayo Elbow Performance Score (MEPS) (a score of 100). Based on our knowledge, the infection should not be a consequence of sequelae. The patients were followed for at least 40 days post operation to evaluate their elbow functional recovery status. Based on the Flynn criteria for elbow evaluation (7), the recovery rate of elbow joint after surgery achieved similar efficiency for the two groups (Figure 3A). Over 90% of the patients lost <10° of both the carrying angle and elbow motion, i.e., classified as excellent or good in the Flynn scheme in both groups. We also assessed patients' postoperative elbow function using the MEPS system and revealed no statistical difference between the two groups (89 ± 10 vs. 91 ± 9, *P* = 0.352) (Figure 3B).

## Discussion

The semi-sterile technique for CRPP is first introduced by a study carried out at the Miami Children's Hospital as a cost-effective alternative for the sterile technique (4). Another recent study confirms the safety and benefits of this technique on the CRPP of upper extremity fractures (3). The sterile technique for supracondylar humerus fracture requires a scrub preparation tray, surgical drapes and sterile gowns, all of which dramatically increases the overall costs of the surgical procedure. In addition, preparation and disposal of these medical materials require further labor costs of health care delivery. Usage of the semi-sterile technique as a cost-effective substitute, where drapes and sterile gowns are not used, not only saves on the economic and labor costs, but also contributes to environmental protection by reducing the generation of medical wastes. In the present retrospective study, we build on top of the previous studies to specifically compare the semi-sterile and the sterile techniques for CRPP from multiple aspects in supracondylar humerus fracture patients, including time, costs and recovery status. A total of 137 patients participated in the study and was separated into the semi-sterile and sterile groups. Overall, application of the semi-sterile technique led to a 75% decrease in the draping and disinfection time. Since the semi-sterile technique only involves a single brush of chlorhexidine and skips the usage of sterile drape, it is reasonable that this parameter was dramatically reduced. In addition, a 17 and 7% reduction in anesthesia time and anesthesia cost, respectively; and an 88% reduction in medical waste cost were also observed in the semi-sterile group. The percentage of reduced anesthesia time is similar to a previous study performed at the SUNY Downstate Medical Center (17 vs. 14%) (3). Considering the high incidence rate of supracondylar humerus fracture among all elbow injuries (8–10), such savings in time and cash may make a big impact on improving the operation turnover rate and reducing the surgical running costs. Intriguingly, no statistical difference was observed in term of the operation time, which does not agree with the previous study where they observed a marginally statistically significant difference (*P* = 0.04) between the two groups (3). This might be explained by the difference in the actual operating procedure rather than the sterile technique.

One of the common complications associated with CRPP is infection (11–14). Superficial pin track infections are normally treated with oral antibiotics, whereas more severe infections, such as arthritis and osteomyelitis, require more robust treatment with intravenous antibiotics and surgical drainage. The overall infection rate is relatively low for CRPP at around 2.34% [summarized in (4)]. It is believed that such low infection rate is due to the relatively short pinning duration required for the recovery (15). In the present study, there was only one superficial infection incidence among the 137 patients. It occurred to a 3-year-old female patient from the semi-sterile group. She suffered from a type 3 supracondylar humerus fracture without any nerve injuries. Because of the rarity of this complication and our small number of patient population, we cannot conclude whether this isolated case is due to the semi-sterile technique or not. The infection was successfully managed by oral admission of antibiotics and the recovered elbow was scored excellent in the Flynn scheme. The only poor-scored recovery was also from the semi-sterile group. An 11-year-old male patient suffered from type 3 supracondylar humerus fracture without nerve injuries lost over 15° of carrying angle and elbow motion after CRPP. This is likely not due to the semi-sterile technique, but secondary to the severity of the injury at the first place or the imperfect reduction of the fracture. It is worth stating: A survey of all the surgeons involved in the treatment of these patients showed that all of them felt safe and comfortable under the semi-sterile conditions and felt that they had no impact of their performance or on the outcome of the procedure.

The present study has several limitations. First, since it is a retrospective study, the assessed parameters are largely dependent on the quality of the data documentation. Intraoperative parameters, such as anesthesia time and operation time, and preparation parameters, such as room setup time and disinfection time, were recorded by different operating room staff and nurses, respectively. Therefore, variations might exist between values recorded by different people. Second, the present study was performed at a single hospital, which limits us to estimate the exact positive financial, time and labor impact the semi-sterile technique can generate on the treatment of pediatric supracondylar humerus fracture. Although our results are generally consistent with a similar previous study carried out at the SUNY Downstate Medical Center (3), more multi-centered studies are required to further uncover the benefits of the semi-sterile technique. Specifically, it is worth noting that the number of patients within the semi-sterile group is relatively limited compared to previous study (4). Therefore, it might not reflect the actual risk percentage of the semi-sterile method. Third, we want to point out that there may have been a selection bias on certain patients for the sterile technique due to the preoperative concern on their fracture status.

## Conclusion

In summary, we confirm that the semi-sterile technique for CRPP utilized in our supracondylar humerus fractured patients greatly reduced the time and costs of the disinfection and anesthesia procedures, without showing any significant increase in post-operative infections. More importantly, the functional recovery status is also not affected by this technique. Our study strongly supports the semi-sterile technique as an alternative to the full-prep sterile technique in CPRR to save operation time and costs. It also provides an indication on its usage in other bone-related surgical procedures.

## Data Availability Statement

All datasets generated for this study are included in the article/supplementary material.

## Ethics Statement

The studies involving human participants were reviewed and approved by ethical committee of Inner Mongolia Autonomous Region. Written informed consent to participate in this study was provided by the participants' legal guardian/next of kin.

## Author Contributions

G-QW and Q-FW performed the analysis. X-DW designed the study and wrote the manuscript. All authors contributed to the article and approved the submitted version.

## Conflict of Interest

The authors declare that the research was conducted in the absence of any commercial or financial relationships that could be construed as a potential conflict of interest.

## Abbreviations

CRPP: closed reduction and percutaneous pinning.
