# Corrigendum: A Comprehensive Comparison Between the Semi-sterile and Sterile Technique for Closed Reduction and Percutaneous Pinning of Pediatric Supracondylar Humerus Fractures

**DOI:** 10.3389/fsurg.2020.640826

**Published:** 2021-01-15

**Authors:** Guo-Qiang Wang, Qing-Feng Wang, Xiao-Dong Wang

**Affiliations:** ^1^Children's Hospital of Soochow University, Suzhou, China; ^2^Department of Orthopedic Surgery, People's Hospital of Ningxia Hui Autonomous Region, Yinchuan, China; ^3^Department of Orthopedics, Children's Hospital of Soochow University, Suzhou, China

**Keywords:** pediatric, sterile, fracture, pinning, recovery, infection

In the original article, there was an error. ^**^There is a mistake in the first paragraph of the Method section, where the hospital name that the patients were treated in is incorrect.^**^

A correction has been made to ^**^Method^**^, ^**^Paragraph 1, first sentence^**^:

**Original text: Upon approval by the review board, a consecutive retrospective cohort study was conducted on pediatric patients who underwent CRPP for supracondylar humerus fracture over a 3-year period (2017-2019) at Children's Hospital of Soochow University**.

**CORRECTED text: Upon approval by the review board, a consecutive retrospective cohort study was conducted on pediatric patients who underwent CRPP for supracondylar humerus fracture over a 3-year period (2017–2019) at Children's Hospital of Bao Tou**.

In the original article, there was an error. ^**^There is a mistake in the first paragraph of the Ethics Statement section, where the name of the approval ethical committee is incorrect.^**^

A correction has been made to ^**^ ETHICS STATEMENT^**^, ^**^Paragraph 1, sentence 1^**^:

**Original text: The studies involving human participants were reviewed and approved by ethical committee of JiangSu province**.

**CORRECTED text: The studies involving human participants were reviewed and approved by ethical committee of Inner Mongolia Autonomous Region**.

The authors apologize for this error and state that this does not change the scientific conclusions of the article in any way. The original article has been updated.

